# MASCC/ISOO Clinical Practice Statement: Adjuvant bone-modifying agents in primary breast cancer patients - prevention of medication-related osteonecrosis of the jaw

**DOI:** 10.1007/s00520-024-08687-w

**Published:** 2024-07-25

**Authors:** Noam Yarom, Catherine H. Van Poznak, Joel B. Epstein, Giulia Ottaviani, Yuhei Matsuda, Cesar Migliorati, Sharon Elad

**Affiliations:** 1 Oral Medicine Unit, Sheba Medical Center, 5265601 Tel Hashomer, Israel; 2 The Maurice and Gabriela Goldschleger School of Dental Medicine, Faculty of Health and Medical Sciences, Tel Aviv University, Tel Aviv, Israel; 3 Department of Internal Medicine, University of Michigan, Ann Arbor, MI USA; 4 Dental Oncology Services, City of Hope National Medical Center, Duarte, CA USA; 5 Cedars Sinai Health System, Los Angeles, CA USA; 6 Department of Medicine, Surgery and Health Sciences, University of Trieste, Trieste, Italy; 7 Department of Oral and Maxillofacial Surgery, Faculty of Medicine, Shimane University, Izumo, Japan; 8 Oral and Maxillofacial Diagnostic Sciences, Oral Medicine, University of Florida College of Dentistry, Gainesville, FL USA; 9 Oral Medicine, Eastman Institute for Oral Health, University of Rochester Medical Center, Rochester, NY USA

**Keywords:** Osteonecrosis of the jaw, Bone-modifying agents, Bisphosphonates, Denosumab, Adjuvant therapy

## Abstract

**Purpose:**

A MASCC/ISOO Clinical Practice Statement (CPS) is aimed at generating a concise tool for clinicians that concentrates practical information needed for the management of oral complications of cancer patients. This CPS raises awareness to the prevention of medication-related osteonecrosis of the jaw (MRONJ) in patients with breast cancer treated with adjuvant bone-modifying agents (BMA).

**Methods:**

This CPS was developed based on a critical evaluation of the literature followed by a structured discussion of a group of leading experts, members of the Oral Care Study Group of MASCC/ISOO. The information is presented in the form of succinct bullets and tables to generate a short manual about the best standard of care.

**Results:**

In patients treated with adjuvant BMA, dento-alveolar surgery poses a moderate risk for MRONJ that ranges between the high risk for MRONJ in patients with metastatic breast cancer and the low risk for MRONJ in patients with osteoporosis. Existing MRONJ guidelines serve as a starting point for adjuvant BMA use. Urgent procedures should be delivered without delay using the accepted precautions to prevent MRONJ. If elective surgery is considered, the individual risk for MRONJ following surgery should be assessed according to common risk factors.

**Conclusion:**

Prevention of MRONJ in primary breast cancer patients treated with adjuvant BMA requires risk–benefit assessment; collaboration between the medical team, dental professional, and patient; and patient-specific tailored dental treatment planning. The patient should be informed about this risk. Additional research is needed to define optimal MRONJ care for this population.

**Supplementary Information:**

The online version contains supplementary material available at 10.1007/s00520-024-08687-w.

## Introduction

Treatment with bone-modifying agents (BMA) such as bisphosphonates (BP) and denosumab is associated with a risk of medication-related osteonecrosis of the jaw (MRONJ) [1]. Even with proper management, MRONJ may persist and affect quality of life and oral function [2]. Different patient populations may have a variable risk for MRONJ [2].

Adjuvant BP therapy is used in patients with non-metastatic breast cancer and correlates with a modest improvement in overall survival [3]. A recent joint guidelines paper from the American Society of Clinical Oncology (ASCO) and Ontario Health (OH) discusses the use of adjuvant BP for all primary breast cancer patients who are postmenopausal (natural or therapy-induced) [4]. Breast cancer affects approximately 300,000 new patients per year in the USA [5] and about 2,000,000 globally [6], and the majority are postmenopausal; therefore, the use of adjuvant BP may increase the incidence of MRONJ.

According to the ASCO/OH guidelines, the most robust evidence supports the following therapeutic options (listed alphabetically):

- Clodronate—1600 mg per os daily for 2–3 years
- Ibandronate—50 mg per os daily for 3 years
- Zoledronic acid (zoledronate, ZA)—4 mg intravenous once every 6 months for 3 years
- ZA—4 mg intravenous once every 3 months for 2 years

To date, there is no formal society recommendation supporting the use of denosumab for this indication. Furthermore, in current studies of denosumab for adjuvant therapy in primary breast cancer, the dose used was either equivalent to osteoporosis dose or to bone metastasis dose [7]. Both doses are addressed in existing guidelines [1, 2]. Therefore, this paper will only refer to BP.

The target population of adjuvant BP is being treated for cure, and these patients are expected to have a long life expectancy compared to metastatic breast cancer. Thus, dentists increasingly will be called upon to treat these patients and the medical team needs to be aware of possible implications on the patient’s future dental care.

In two out of the four ASCO/OH BP regimens, the recommended dose for adjuvant BP in patients with primary breast cancer is similar to the dose recommended for metastatic breast cancer (clodronate and ibandronate regimens; see Table 1). Therefore, these patients treated with the ibandronate regimen should be addressed according to the existing guidelines for dental management of metastatic breast cancer patients [1, 2]. Clodronate is not in common use nowadays or not approved for use in many countries, including in the USA. Accordingly, ibandronate and clodronate are excluded from the discussion in this publication. Notwithstanding, the exclusion of ibandronate and clodronate does not suggest that they are not associated with increased risk for MRONJ.

**Table 1:** Common BP regimens in osteoporosis, metastatic cancer, multiple myeloma, and primary cancer

	Osteoporosis		Bone metastases		Adjuvant therapy (primary breast cancer)	
	Regimen	Estimated annual dose (mg)	Regimen	Estimated annual dose (mg)	Regimen	Estimated annual dose (mg)
Clodronate *			PO 1600 mg/d	584,000	PO 1600 mg/d, for 2–3 yr	584,000
Ibandronate	PO 150 mg/mo	1800	PO 50 mg/d *	18,250	PO 50 mg/d, for 3 yr *	18,250
Zoledronate	IV 5 mg/yr	5	IV 4 mg/mo	48	IV 4 mg/6 mo, for 3 yr	8
			IV 4 mg/3 mo	16	IV 4 mg/3 mo, for 2 yr	16

^*^Not available in the USA; *PO*, per os; *IV*, intravenous; *mo*, month; *d*, day; *yr*, years

In the remaining two ASCO/OH BP regimens (ZA regimens; see Table 1), the annual dose of this BP (8 mg/year or 16 mg/year) is lower than the annual dose of this BP in metastatic disease (16–48 mg/year). Furthermore, these regimens for the adjuvant ZA in primary breast cancer are administered for a limited duration (2–3 years) relative to the BP regimens in metastatic cancer which are often longer (typically indefinite) [8]. Therefore, the cumulative dose is expected to be lower in primary breast cancer administered adjuvant ZA. Accordingly, this CPS will focus on the adjuvant ZA regimens for primary breast cancer, which may have new implications related to MRONJ.

In 2019, the Multinational Association of Supportive Care in Cancer (MASCC), the International Society of Oral Oncology (ISOO), and the American Society of Clinical Oncology (ASCO) published clinical practice guidelines for the prevention and management of MRONJ in cancer patients [1]. This comprehensive publication focused on patients with metastatic cancer or multiple myeloma, and did not refer to patients treated with adjuvant BP. Evidence regarding MRONJ in patients with primary breast cancer is scarce resulting in a clinical gap regarding the prevention of MRONJ in patients treated with adjuvant BP. There are two unique aspects related to this patient population—dental care before and during adjuvant BP and long-term dental care. As patients age, the need for complex dental procedure may increase, and the risk for MRONJ is higher with invasive interventions. Conversely, the risk for MRONJ may decrease after the cessation of the adjuvant BP. Therefore, a working group of the Oral Care Study Group (OCSG) of MASCC/ISOO developed this clinical practice statement (CPS) to suggest an approach to patients with primary breast cancer treated with BP in the adjuvant setting.

## Objectives

This study aims to raise awareness to the risk for MRONJ in patients with breast cancer treated with adjuvant ZA, to provide risk-mitigating strategies, and, additionally, to outline the main considerations in the dental management of these patients in order to prevent MRONJ.

## Methods

This CPS is based on a compilation of expert opinions with a high-quality review of the literature. The literature search was conducted on PubMed on data pertinent to MRONJ and adjuvant BP in the timeframe up to January 1, 2023. During the development of the manuscript, point questions that deemed a closer look were generated, and a literature search was done to ensure the accuracy of the information. The CPS was discussed internally by a working group of OCSG members who are experts on the topic of MRONJ, and then reviewed by two independent boards: the ISOO Advisory Board and the MASCC Guidelines Committee. The Statement follows the MASCC/ISOO Guidelines Policy.

## Clinical relevance and practical considerations

- Multi-disciplinary coordination and patient education
  - Dental care should be coordinated between the dentist and the oncology team to ensure that necessary procedures are undertaken prior to the initiation of adjuvant BP. An open discussion between the patient, the oncologist, and the dentist regarding the risks, benefits, and extent of the prophylactic dental treatment is advised in order to achieve optimal care and a better quality of life. As there is no information if delay in starting adjuvant BP decreases its efficacy in the prevention of metastases, delay of dental care should be minimal. ASCO/OH suggests initiating BP within 3 months of definitive surgery or within 2 months of completion of adjuvant chemotherapy [4].
  - Given that the benefit of adjuvant BP treatment to the patient’s overall survival is modest [4], the value of prescribing the adjuvant BP should be weighed relative to the likelihood for future invasive dental procedures within the immediate and long-term time period.
  - Patients should be educated regarding the risk for MRONJ (Table 2) and the importance of dental evaluation prior to adjuvant BP treatment and thereafter.

**Table 2:** Rate of MRONJ in adjuvant bisphosphonate protocols for primary breast cancer

Bisphosphonate	Author, year	Protocol name	Protocol	Study population	Rate of MRONJ *
Clodronate	Gralow 2020 [9]	SWOG S0307	PO 1600 mg/d, for 3 yr	2268	0.36%
Ibandronate	Gralow 2020 [9]	SWOG S0307	PO 50 mg/d, for 3 yr	1552	0.77%
Zoledronic acid	Gralow 2020 [9]	SWOG S0307	Step 1: IV 4 mg/mo, for 6 mo Step 2: IV 4 mg/3 mo, for 2.5 yr	2231	1.26%
	Gnant 2011 [10]	ABCSG-12	IV 4 mg/6 mo, for 3 yr	1803	0%
	Perrone 2019 [11]	HOBOE	IV 4 mg/6 mo, for 5 yr	1065	1.12%
	Friedl 2021 [12]	SUCCESS	Step 1: IV 4 mg/3 mo, for 2 yr Step 2: IV 4 mg/6 mo, for 3 yr	1540	0.71%
			IV 4 mg/3 mo, for 2 yr	1447	0.34%
	Coleman 2018 [13]	AZURE	Step 1: IV 4 mg/mo, for 6 mo Step 2: IV 4 mg/3 mo, for 2 yr Step 3: IV 4 mg/6 mo, for 2.5 yr	3360	1.8%

^*^The proportion of patients developing MRONJ following dento-alveolar surgical procedure vs. patients developing MRONJ spontaneously is unknown

*MRONJ*, medication-related osteonecrosis of the jaw; *mo*, months; *yr*, years; *IV*, intravenous; *PO*, per os
  - The oncology care team should advise the patients to inform the dentist about being treated with BP, either planned, current, or prior doses, regardless of the type of dental procedure. This is of utmost importance, since patients may omit reporting treatments given intravenously in an ambulatory care setting biannually. This communication augments the information flow about all other comorbidities and medication lists. The dental care team should be aware that patients with non-metastatic breast cancer may be treated with BP.
  - The dental care team should advise the patients to have routine dental check-ups and periodontal maintenance throughout the adjuvant BP treatment and following its completion. Patients should be encouraged to practice meticulous daily oral hygiene as a preventive measure. For more details on the information that should be delivered to patients prior to BP therapy, please see Table 4 in the MASCC/ISOO/ASCO guidelines paper [1].
  - Patients are advised to consult with an experienced dental specialist prior to high-risk dento-alveolar procedures or when in doubt about the dental treatment planning.
- Treatment plan considerations
  - Dental treatment prior to the initiation of adjuvant BP should be individualized according to the patient’s risk factors. The presence of a likely risk factor for MRONJ (such as steroid use) may drive a more deterministic dento-alveolar surgical approach prior to the initiation of the adjuvant BP [2]. Other possible risk factors for MRONJ that have inconsistent evidence include diabetes, age, smoking, anemia, and certain concurrent medications, such as angiogenesis inhibitors, periodontal disease, and the presence of dentures [1, 2, 14, 15]. Accumulating dose of BP should also play a role in the risk assessment [2]. Therefore, extension of the adjuvant BP beyond the ASCO recommendation or BMA treatment prior to the adjuvant BP for osteoporosis poses an additional risk.
  - Given the lower annual cumulative dose of adjuvant ZA in primary breast cancer patients (8 or 16 mg/year) relative to ZA dose in metastatic breast cancer (16–48 mg/year), the risk for MRONJ is likely lower in the former group. On the other hand, the risk for MRONJ with the adjuvant BP regimen is probably higher than in osteoporosis patients (5 mg/year) [16]. Therefore, the clinical approach for dento-alveolar surgery should be at a mid-level strategy, and consider the extent of total exposure of ZA.
  - This patient population may be treated with additional pharmacological agents. As new therapies are introduced in the market and the profile of their adverse effects is being revealed, modifications to the dental treatment plan may be needed. In particular, the clinicians should be aware of the patient’s immune status and possible risk for oral infections.
  - For more details on the dental preparations of patients prior to BP therapy, please see Table 3 in the MASCC/ISOO/ASCO guidelines paper [1].
  - Geographic variation in health and or dental insurance may impact the implementation of this CPS. Attempts should be made to address dental needs within the economic challenges.
- Dento-alveolar surgery for patients on adjuvant BP
  - Urgent procedures should be delivered without delay. Antibiotics should be considered if signs of infection/inflammation are observed. Standard postoperative practices to prevent secondary infections should be employed.
  - The evidence of the risk for MRNOJ following a dento-alveolar surgery in adjuvant BP regimens is scarce. It is unknown whether elective procedures should be delivered during the 2–3 years of the adjuvant BP regimens. Therefore, the clinician should consider the necessity of dento-alveolar procedures carefully.
  - Elective dento-alveolar surgery in patients taking adjuvant BP is estimated to pose a moderate risk for MRONJ that ranges between the high risk for MRONJ in patients with metastatic breast cancer and the low risk for MRONJ in patients with osteoporosis.
  - In elective dento-alveolar surgery, the risk should be stratified individually according to common risk factors (see Box).
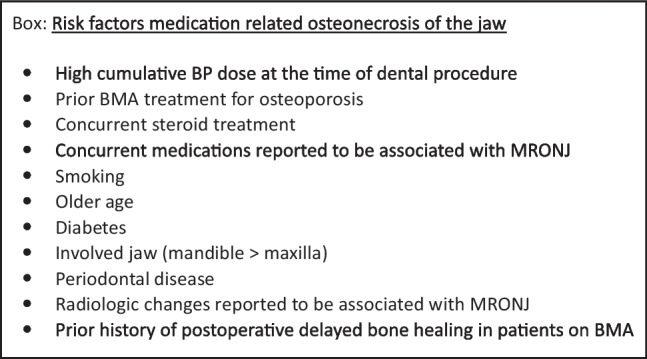
  - In patients with a higher cumulative dose of BP or multiple risk factors for MRONJ, a more conservative dento-alveolar surgical approach is advised, as in patient taking the ZA dose for metastatic cancer [2].
  - The optimal timing of an elective bone invasive procedure in relationship to BP dosing is unknown. If a dento-alveolar surgery is deemed necessary, and in order to improve wound healing and decrease the risk for MRONJ, it is speculated that it is preferable to:
    - Perform the procedure as far as possible from the last BP dose.
    - After the dento-alveolar procedure, hold the next BP dose until adequate osseous healing is observed.
  - For more details on the considerations related to surgical site manipulation, please see the AAOMS position paper [2].
- Dento-alveolar surgery for patients who completed adjuvant BP
  - There are insufficient data at this time to allow a recommendation. The risk for MRONJ may decrease after the completion of adjuvant BP; however, as the clinical experience and literature increase, the clinical approach will get clear.
- Management
  - If MRONJ develops in this patient population, it should be managed as per established guidelines [1, 2].
  - It is advised to follow up for updates in the literature, as new information on MRONJ management evolves.

## Supplementary Information

Below is the link to the electronic supplementary material.

Supplementary file1 (DOCX 18.5 KB)

## Data Availability

No datasets were generated or analyzed during the current study.

## Declarations

### Disclaimer

The MASCC/ISOO OCSG Statements have been developed to facilitate expert opinion-based management of oral complications of cancer and cancer therapy, where high-quality evidence is lacking. Clinicians should use their judgment when making treatment decisions for individual patients. Statement authors and the MASCC/ISOO do not guarantee or take responsibility for the clinical outcomes in individual patients.
