# Supplementary material for: MASCC/ISOO Clinical Practice Statement: Adjuvant bone-modifying agents in primary breast cancer patients - prevention of medication-related osteonecrosis of the jaw
Source: Support Care Cancer. 2024 Jul 25;32(8):547. doi: 10.1007/s00520-024-08687-w (PMC11269502; doi:10.1007/s00520-024-08687-w)
Supplement: Supplementary file 1 — Supplementary file1 (DOCX 18.5 KB) [file 520_2024_8687_MOESM1_ESM.docx]

**MASCC/ISOO Clinical Practice Statement: adjuvant bone-modifying agents in primary breast cancer patients — prevention of medication-related osteonecrosis of the jaw**

**Suggested reading:**

1. Coleman R, Finkelstein DM, Barrios C, Martin M, Iwata H, Hegg R, Glaspy J, Periañez AM, Tonkin K, Deleu I, Sohn J, Crown J, Delaloge S, Dai T, Zhou Y, Jandial D, Chan A. Adjuvant denosumab in early breast cancer (D-CARE): an international, multicentre, randomised, controlled, phase 3 trial. Lancet Oncol. 2020 Jan;21(1):60-72. doi: 10.1016/S1470-2045(19)30687-4. Epub 2019 Dec 2. PMID: 31806543.
2. McGee S, AlZahrani M, Stober C, Ng TL, Cole K, Larocque G, Awan A, Sehdev S, Hilton J, Vandermeer L, Hutton B, Pond G, Saunders D, Clemons M. Adjuvant bisphosphonate use in patients with early stage breast cancer: Patient perspectives on treatment acceptability and potential de-escalation. J Bone Oncol. 2021 Feb 19;27:100351. doi: 10.1016/j.jbo.2021.100351. PMID: 33680749; PMCID: PMC7930351.
3. McGee S, Alzahrani M, Vandermeer L, Cole K, Larocque G, Awan A, Hutton B, Pond G, Saunders D, Clemons M. Adjuvant bisphosphonate use in patients with early stage breast cancer: a physician survey. Breast Cancer Res Treat. 2021 Jun;187(2):477-486. doi: 10.1007/s10549-021-06147-1. Epub 2021 Mar 23. PMID: 33755864; PMCID: PMC7985746.
4. Peng J, Wang H, Liu Z, Xu ZL, Wang MX, Chen QM, Wu ML, Ren XL, Liang QH, Liu FP, Ban B. Real-world study of antiresorptive-related osteonecrosis of jaw based on the US food and drug administration adverse event reporting system database. Front Pharmacol. 2022 Oct 19;13:1017391. doi: 10.3389/fphar.2022.1017391. PMID: 36339548; PMCID: PMC9627332.
5. Shapiro CL. Bone-modifying Agents (BMAs) in Breast Cancer. Clin Breast Cancer. 2021 Oct;21(5):e618-e630. Doi: 10.1016/j.clbc.2021.04.009. Epub 2021 Apr 26. PMID: 34045175.
6. Beltran-Bless AA, Clemons MJ, Fesl C, Greil R, Pond GR, Balic M, Vandermeer L, Bjelic-Radisic V, Singer CF, Steger GG, Helfgott R, Egle D, Sölkner L, Gampenrieder SP, Kacerovsky-Strobl S, Suppan C, Ritter M, Rinnerthaler G, Pfeiler G, Fohler H, Hlauschek D, Hilton J, Gnant M. Does the number of 6-monthly adjuvant zoledronate infusions received affect treatment efficacy for early breast cancer? A sub-study of ABCSG-12. Eur J Cancer. 2023 Feb;180:108-116. Doi: 10.1016/j.ejca.2022.12.003. Epub 2022 Dec 10. PMID: 36592505.
7. Mittal A, Tamimi F, Molto C, Di Iorio M, Amir E. Benefit of adjuvant bisphosphonates in early breast cancer treated with contemporary systemic therapy: A meta-analysis of randomized control trials. Heliyon. 2024 Jan 19;10(2):e24793. doi: 10.1016/j.heliyon.2024.e24793. PMID: 38312616; PMCID: PMC10835314.
8. Abdolrahmani A, Epstein JB, Samim F. Medication-related osteonecrosis of the jaw: evolving research for multimodality medical management. Support Care Cancer. 2024 Mar 5;32(4):212. doi: 10.1007/s00520-024-08388-4. PMID: 38443685.
